# Cleaning Teeth Reduces the Inflammatory Response of Macrophages to Acid Dentine Lysate

**DOI:** 10.3390/ijms21239207

**Published:** 2020-12-02

**Authors:** Jila Nasirzade, Zahra Kargarpour, Layla Panahipour, Frank Schwarz, Reinhard Gruber

**Affiliations:** 1 Department of Oral Biology, Medical University of Vienna, 1090 Vienna, Austria; jila.nasirzaderajiri@meduniwien.ac.at (J.N.); zahra.kargarpooresfahani@meduniwien.ac.at (Z.K.); layla.panahipour@meduniwien.ac.at (L.P.); 2 Department of Oral Surgery and Implantology, Johann Wolfgang Goethe-University, Carolinum, 60596 Frankfurt, Germany; f.schwarz@med.uni-frankfurt.de; 3 Department of Periodontology, School of Dental Medicine, University of Bern, 3012 Bern, Switzerland; 4 Austrian Cluster for Tissue Regeneration, 1200 Vienna, Austria

**Keywords:** autograft, tooth, transplantation, augmentation, dentistry, inflammation, macrophages, LPS, acid dentine lysate, cleaning

## Abstract

Particulate autogenous tooth roots are used for alveolar bone augmentation surgery; however, dental plaque may provoke an inflammatory response that may counteract the desired graft consolidation process. Traditional mechanical cleaning of extracted teeth may be of support to lower a possible inflammatory response of the autograft. To test this assumption, extracted porcine teeth were left either uncleaned or underwent mechanical cleaning with a toothbrush and toothpaste before being fragmented and subjected to acid lysis, termed as unclean acid dentine lysate (ucADL) and clean acid dentine lysate (cADL), respectively. The inflammatory responses of murine macrophage RAW 264.7 cells being exposed to the respective acid dentine lysates were evaluated at the level of inflammatory gene expression and IL6 immunoassays. We report here that acid lysates obtained from uncleaned teeth provoked a robust increase in IL1β, IL6, and COX2 in RAW 264.7 cells. The mechanical removal of dental plaque significantly reduced the inflammatory response. Consistently, Limulus tests revealed that tooth cleaning lowers the presence of endotoxins in dentine lysates. To further prove the involvement of endotoxins, a toll-like receptor 4 (TLR4) inhibitor TAK242 was introduced. TAK242 abolished the inflammatory response provoked by acid lysates obtained from uncleaned teeth in RAW 264.7 cells. Moreover, nuclear translocation and phosphorylation of the TLR4 downstream NFκB-p65 were attenuated at the presence of cleaned versus uncleaned dentine lysates. Taken together, our data support the importance of dental plaque removal of teeth being extracted for alveolar bone augmentation surgery.

## 1. Introduction

Autologous tooth roots have gained increasing attention for oral bone augmentation [1] and were systematically investigated for lateral augmentation in deficient extraction sockets [2] and ridges prior to implant placement [3]. This clinical concept is based on radiological analyses [4,5], case reports [6,7] and preclinical studies [8,9,10]. These studies support the clinical use of autologous tooth roots and particularly dentine as a graft material for bone augmentation. The clinical principle is based on the similarities of dentine and bone; both being mineralized tissues, osteoconductive and subjected to bone remodeling during the course of graft consolidation. Thus, dentine in the same way as bone serves as a grafting material; however, in contrast to bone that is protected by the soft tissue, the tooth crown and potentially also parts of the root dentine are exposed to the oral cavity [2,7]. It can thus not be ruled out that at least a contaminating microbiome is co-transplanted with the teeth roots [11,12].

The microbiome of the oral cavity is complex and so is the biofilm that firmly attaches to the tooth surface, in particular when oral hygiene is neglected [11,12]. Apart from oral hygiene, it is that the increasingly recognized impact of diet, nutrition and nutraceuticals on oral and periodontal health [13]. For example, neuridase, a food supplement based on palmitoylethanolamide and dry extract of the roots of *Scutellaria baicalensis*, can potentially affect the oral microbiome and reduce local inflammation [14]. The changing microbiom can be identified by salivary immunoglobulins raised against A. actinomycetemcomitans [15], and may even affect the plasminogen activator system linking oral health with cardiovascular disease [16]. It is thus not surprising that saliva holds a potent pro-inflammatory capacity for various cell types including macrophages [17], and that the biofilm or plaque proceeding into a hard calculus is the main trigger of chronic inflammation in the oral cavity. It can be assumed that erupted teeth considered as graft material are carefully cleaned but possibly contaminated by the remaining biofilm. This is the reason why the effect of autoclavation on the efficacy of extracted tooth roots used for vertical alveolar ridge augmentation was investigated [1]. The endotoxins of the biofilm causing the inflammatory response are, however, resistant against heating and it is maybe the traditional mechanical tooth cleaning with a toothbrush and toothpaste that lowers the endotoxin load. 

Endotoxins, particular lipopolysaccharides (LPS) being large lipids bound to polysaccharides, are produced by gram negative bacteria [18]. LPS is a major trigger of an inflammatory response that is mediated via the pattern recognition receptors [19]. It is mainly the toll-like receptor 4 (TLR4) complex expressed in cells of the myeloid lineage that binds LPS [20]. TLR4 became a pharmacologic target that led to the development of inhibitors including TAK242 [21], also blocking saliva-induced macrophage activation in vitro [17]. RAW 264.7 mouse macrophage as well as primary murine bone marrow cultures are highly sensitive to saliva and therefore sensitive bioassays for LPS in autologous tooth roots. However, it requires the processing of tooth roots to isolate LPS. Here, we used acid dentine lysates (ADL), similar to what we have introduced, to prepare acid bone lysates [22,23,24]. 

With respect to the aforementioned background, the study tries to evaluate the hypothesis that cleaning of the tooth lowers down inflammatory response at the augmentation site. Therefore, the first aim of this research is to show that acid dentine lysates prepared from freshly extracted porcine teeth provoke an in vitro inflammatory response in macrophages. The second aim is to test if traditional mechanical tooth cleaning with a toothbrush and toothpaste can decrease the inflammatory response. Thus, the overall goal of this research is to support the use of traditional mechanical tooth cleaning with a toothbrush and toothpaste prior to the processing of extracted teeth into autologous tooth roots for bone augmentation.

## 2. Results

### 2.1. ADL Increases Production of Formazan in Raw 264.7

**Figure 1:**
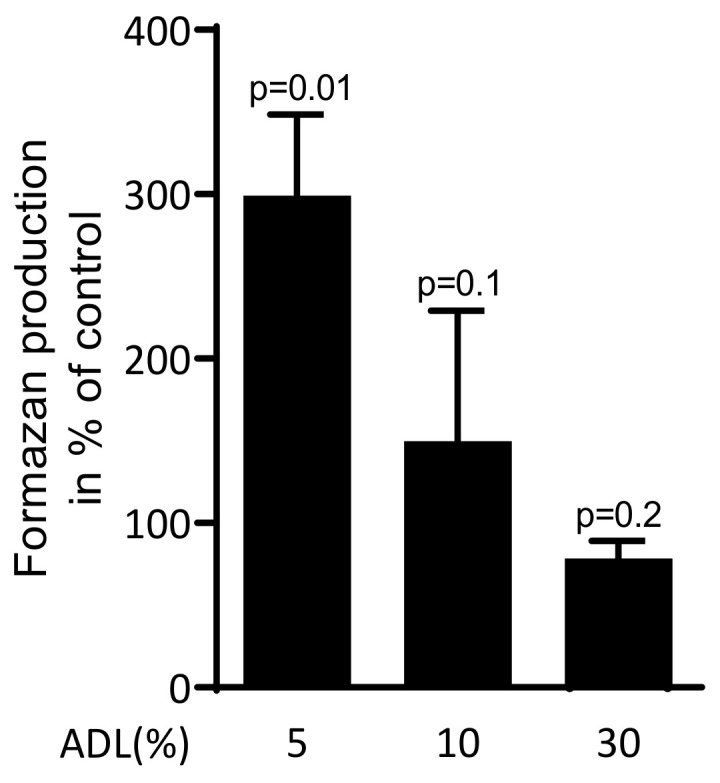
Acid dentine lysates (ADL) maintain viability of RAW 264.7 cells.

To investigate the effect of ADL on viability of RAW 264.7 cells, the reduction of the tetrazolium dye into to insoluble formazan was measured. ADL at 5% significantly increased the formazan production. At 10% ADL, however, the production of formazan was similar compared to untreated cells (Figure 1). Thus, ADL was applied at 5% where RAW 264.7 cells were even supported in their activity. 

The viability of macrophage-like RAW 264.7 cells was estimated using MTT assay. RAW 264.7 cells were exposed to 5%, 10% and 30% of ADL. The optical density of formazan produced by cells was measured compared to unstimulated control (100%). Data represent the mean ± SD of three independent experiments relative to the unstimulated control. Statistic is based on the Kruskal–Wallis test.

### 2.2. Blocking of TLR4 Interferes with Endotoxin Activity of Unclean ADL

**Figure 2:**
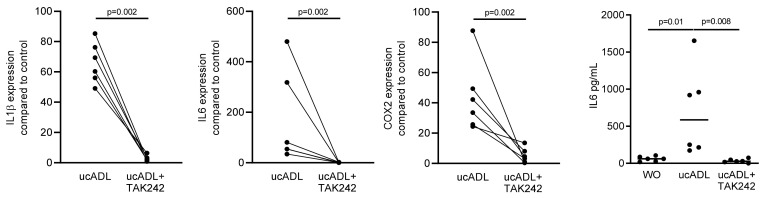
ADL-pro-inflammatory effect is TLR4-dependent.

Considering that dental plaque is a rich source of LPS provoking an inflammatory response of RAW 264.7 cells [25], we determined the inflammatory response of RAW 264.7 cells exposed to ADL from unclean teeth (ucADL) with and without blocking of TLR4 by TAK242. Gene expression analysis showed the expected increased expression of the inflammatory markers IL1β, IL6 and COX2 in RAW 264.7 that was blocked by TAK242 (Figure 2). The same was true for the TAK242-dependent IL6 at the protein level (Figure 2). These results suggest that ADL from uncleaned teeth provokes a TLR4-dependent inflammatory response in macrophages.

RAW 264.7 cells were exposed to ADL from uncleaned teeth (ucADL) with and without TAK242. The gene expression levels of IL1β, IL6, and COX2 were evaluated. Protein level of IL6 was measured. These results show TAK242 significantly blocked the expression of inflammatory markers induced by ADL from uncleaned teeth. Data points represent independent experiments. Statistic was performed by Mann–Whitney test and Friedman test, for gene expression and immunoassay analysis, respectively. 

### 2.3. Removing of Dental Plaque Abolishes the Inflammatory Response of RAW 264.7

**Figure 3:**
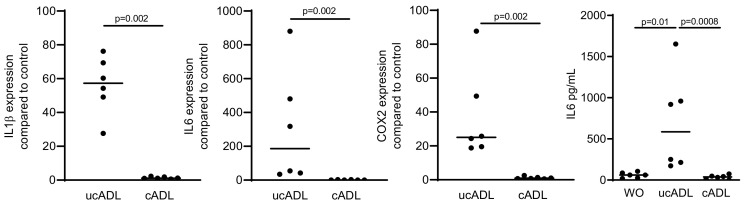
Removing of the dental plaque reduces pro-inflammatory effect of ADL.

We next examined how traditional mechanical tooth cleaning with a toothbrush and toothpaste affects the inflammatory capacity of ADL. Consistent with the ability of tooth cleaning to reduce the dental plaque and LPS [26], the respective ADL had a significant lower capacity to provoke an inflammatory response in RAW 264.7 cells compared to ADL prepared from uncleaned teeth (Figure 3). The data suggest that mechanical tooth cleaning prior to their processing into particles lowers the capacity of ADL to provoke an inflammatory response in macrophages.

Teeth were divided into two groups, one being brushed and cleaned using a toothpaste and another group being left uncleaned. ADL from cleaned and uncleaned teeth (cADL and ucADL, respectively) were used to stimulate RAW 264.7 cells. LPS (100 ng/mL) was used as a positive control. Gene expression levels of IL1β, IL6, COX2, and the protein level of IL6 were evaluated. Cleaning of the teeth reduces the inflammatory effect of ADL produced on RAW 264.7 cells. Data points show independent experiments. Statistic was performed by Mann–Whitney test and Friedman test, for gene expression and immunoassay analysis, respectively. 

### 2.4. Removing of Dental Plaque Drops down the Endotoxin Content of Dentine 

**Figure 4:**
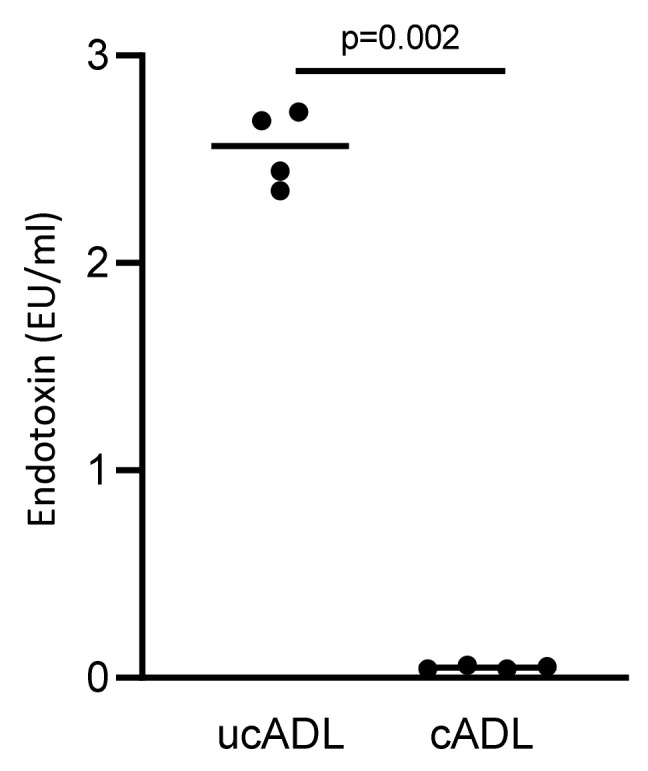
Removing dental plaque reduces the level of endotoxin in ADL.

To further confirm that mechanical tooth cleaning lowers the contamination of ADL with LPS/endotoxin, a LAL test was performed. In support of this assumption, ADL from uncleaned teeth showed a substantial contamination with LPS while ADL from cleaned teeth presented almost no signal in the LAL test (Figure 4). These results confirm that tooth cleaning reduces the dental plaque-derived LPS that is responsible for provoking an TLR-4-mediated inflammatory response in RAW 264.7 cells.

Endotoxin content of ADL derived from clean and unclean teeth were measured using a LAL test. Four independent preparations of clean and uncleaned ADL were evaluated. Cleaning of the teeth substantially reduces the endotoxin content of ADL. Statistic was performed based on Mann–Whitney test.

### 2.5. Clean ADL Does Not Induce Phosphorylation of p65 

**Figure 5:**
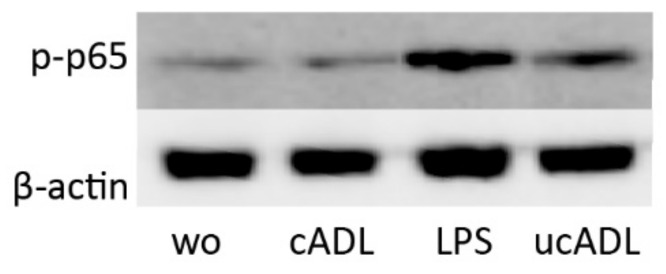
Clean ADL (cADL) does not induce phosphorylation of p65. Phosphorylation of p65 was increased by unclean ADL (ucADL) but less compared to LPS. “wo” stands for without, indicating the unstimulated cells.

To further prove the involvement of NF-κB signaling downstream of TLR4, the phosphorylation of p65 was determined by Western blot analysis. Phosphorylation of p65 was considerably lower in the presence of clean ADL in comparison to ADL from uncleaned teeth (Figure 5). 

### 2.6. Clean ADL Does Not Induce Translocation of p65

**Figure 6:**
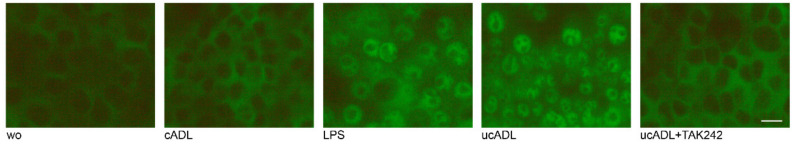
Cleaning of teeth reduces the translocation of p65. RAW 264.7 cells were exposed to ADL from uncleaned teeth (ucADL) with and without TAK242. LPS (100 ng/mL) was used as a positive control. Intracellular translocation of p65 was monitored by immunofluorescence staining. Intracellular translocation of p65 was attenuated in the presence of clean ADL. This was also observed when blocking the TLR4 by TAK242 in cells stimulated by uncleaned ADL. Scale bar indicates 100 μm. “wo” stands for without, indicating the unstimulated cells.

Finally, to further prove the involvement of NF-κB signaling downstream of TLR4, translocation of p65 was determined by immunostaining. Translocation of p65 was obviously induced by ADL from uncleaned teeth but only weak signals were visible when RAW 264.7 cells are exposed to ADL from cleaned teeth (Figure 6). TAK242 blocked the translocation of p65 induced by ADL from uncleaned teeth in RAW 264.7 cells. Thus, tooth cleaning lowers the capacity of ADL to induce the nuclear translocation of p65. 

## 3. Discussion

This research was motivated by the increasing clinical use of autologous tooth roots for oral bone augmentation [1,2,4,5,6]. The tooth crown and root dentine, however, may be contaminated by dental plaque, being a rich source of endotoxins [27]. LPS, being the hallmark endotoxin, can then activate the TLR4 – NF-κB signaling cascade that drives the expression of cytokines and other mediators that in turn initiate or even maintain an inflammatory tissue response [18,19,20]. Considering that LPS reaches the sites of augmentation via the clinical application of autologous tooth roots, easy strategies to lower the contamination with LPS are required, with traditional mechanical tooth cleaning using a toothbrush and toothpaste being the first line approach. In support of this concept, ADL obtained from uncleaned teeth provoked a robust TLR4-NF-κB-mediated increase in inflammatory cytokines and COX2 in a widely established macrophage cell line. More important, however, is that ADL from cleaned teeth has not caused a similar inflammatory response. We therefore conclude that mechanical tooth cleaning is highly effective in removing the endotoxins and thus the cause of the inflammatory response. 

If we relate our findings to those of others we can confirm that uncleaned teeth and thus the presence of a dental plaque hold an endotoxin activity based on our bioassay [27]. In support of previous findings that TAK242 [21] blocks saliva-induced macrophage activation in vitro [17] is that TAK242 also blocks the inflammatory response induced by ADL obtained from uncleaned teeth. Maybe not surprising is the observation that mechanical tooth cleaning removes dental plaques and thereby eliminates the endotoxins causing macrophage activation [28]; however, preparing ADL, similar to what has been shown for acid bone lysates [22,23,24], and testing for the endotoxin activity has not been performed so far. Moreover, the Limulus amoebocyte lysate was used to measure endotoxins in mouth rinses [29]. Taken together, our findings suggest that tooth brushing lowers the plaque-endotoxin-TLR4 activation of macrophages. 

In the clinical scenario, caries-free, partially or fully retained or impacted wisdom teeth without signs of local pathologies are used for auto transplantation. These teeth are not or only marginally contaminated with plaque, as canines, premolars and molars were also used for preparing block grafts [2,7]. Importantly, periodontally diseased tooth roots were applied for lateral alveolar ridge augmentation, at least in a canine model [6]. Periodontitis promotes host inflammatory mediators from the fibroblasts and macrophages in response to bacteria in the biofilms [15]. Histologically, teeth that underwent scaling and root planing were not associated with any inflammatory cell infiltrates [6], consistent with our observations. The existing knowledge, together with our findings, leads to the general assumption that mechanical tooth cleaning, particular when periodontally compromised teeth are used for augmentation, in addition to scaling and root planing [6], may reduce the endotoxin level of the grafted teeth. 

The clinical relevance of our work, however, is compromised by the fact that mechanical tooth cleaning is not a sterile procedure; thus, transplanting these teeth is not feasible. This is obviously a major limitation of our work from a clinical perspective. The current study has further limitations. We could not standardize the level of plaque from the extracted pig teeth, thus not representing the clinical situation prior to a tooth extraction were preferentially clean teeth previously impacted in the jaw bone are used [2,7]. Future studies should include human ADL following the established protocols to rule out any possible contamination with endotoxins [2,7]. We also have not standardized the mechanical cleaning of extracted teeth with a toothbrush and toothpaste, however we try to do the cleaning for at least 15 min. Future research might consider the processing of teeth into ADL to study the impact of scaling and root planing, or taking a burr, on the removing of the dental plaque from periodontally diseased tooth roots used for lateral alveolar ridge augmentation. Based on the concept that diet, nutrition and nutraceuticals can affect oral and periodontal health [13], future research should also consider this aspect in the contexts of plaque accumulation on extracted teeth indicated for bone grafting and the overall process of graft consolidation, which basically depends on the bone regeneration capacity of the patient. 

Taken together, we show here that preparing ADL from freshly extracted pig teeth releases the endotoxins from the dental plaque that can be detected by means of bioassays using the expression of inflammatory cytokines and the traditional limulus test. We have confirmed the activation of macrophages via the TLR4-NFkB signaling pathway. The present research is a further evidence that the mechanical cleaning of extracted teeth with a toothbrush and toothpaste is an efficient procedure to remove the plaque and thus the endotoxins. Even though this research was inspired by the clinical transplantation of autologous teeth, we have to confess that the clinical relevance of the present research is rather theoretical, but nevertheless, it should sensitize clinicians to the possible risk of bacterial endotoxin when maybe periodontally diseased tooth roots are transplanted.

## 4. Materials and Methods 

### 4.1. Acid Dentine Lysate (ADL)

Teeth were extracted from adult pigs within 6 h post-mortem from a local butcher (Fleischerei Leopold Hödl, Vienna, Austria). Following mechanical removing the gingiva with a surgical blade (Swann-Morton, Sheffield, United Kingdom) and the enamel using a manual grinding and polishing device (Metaserv 2000, Cleveland, Ohio), the dental pulp was eradicated with probe (Instrapac, Worksop, United Kingdom). The remaining tooth roots were divided into two groups, one being brushed (R.O.C.S, Tallinn, Estonia) using toothpaste (R.O.C.S, Tallinn, Estonia) for 15 min while the other group remained uncleaned. To increase the surface, a hammer crushed tooth roots that were now supposed to consist of dentine. One gram of wet crushed dentine was incubated while being stirred overnight at room temperature with 10 mL of 0.1 N HCl (10% weight/volume). The dentine lysate was centrifuged and the pH of the supernatant was neutralized. Following sterile filtration, the acid dentine lysates (ADL) were kept frozen at −20 °C. The stocks were thawed immediately before each experiment.

### 4.2. Cell Culture

RAW 264.7 macrophage-like cells (ATCC, Manassas, VA, USA) were expanded in Dulbecco Modified Medium (DMEM) supplemented with 10% fetal bovine serum, antibiotics (all Invitrogen, Grand Island, NY, USA) and seeded 1 × 10^6^ cells/cm^2^ into 96- and 24-well culture plates (VWR, Vienna, Austria). The next day, RAW 264.7 cells were exposed to 5% of ADL with or without TLR4 inhibitor, TAK-242 (Merck Millipore, Billerica, MA, USA) and lipopolysaccharide (LPS) from *Escherichia coli* 0111:B4 at 100 ng/mL (Sigma-Aldrich, St. Louis, MO, USA) for another 24 h under standard conditions at 37 °C, 5% CO_2_, and 95% humidity. Then, viability, RT-PCR and immunoassay were performed. For immunostainings, the protocol was adapted and cell exposure was one hour as stated below. 

### 4.3. Analysis of Formazan Production by MTT Assay

RAW 264.7 cells were seeded on a microtiter plate (CytoOne, Sunnyvale, CA, USA) and exposed to indicated concentrations of ADL for overnight. Following that, RAW 264.7 cells were incubated with 5 mg/mL of MTT (3-[4,5-dimethythiazol-2-yl]-2,5-diphenyltetrazolium bromide; Sigma) for 4 h under culture conditions. Optical density (OD) of DMSO-solubilized formazan crystals were measured at 570 nm. Data were presented as the percentage of OD of stimulated cells normalized to the unstimulated cells.

### 4.4. RT-PCR and Immunoassay

Total RNA of RAW 264.7 cells was extracted (ExtractMe, Blirt S.A., Gdańsk, Poland) and reversely transcripted with cDNA synthesis kit (LabQ, LabConsulting, Vösendorf, Austria Vienna). RT-PCR was performed using the manufacturer’s instructions (LabQ; LabConsulting). Primer sequences were mGAPDH-F: AACTTTGGCATTGTGGAAGG, mGAPDH-R: GGATGCAGGGATGATGTTCT, mIL1β-F: AAGGGTGCTTCCAAACCTTTGAC, mIL1β-R: ATACTGCCTGCCTGAAGCTCTTGT; mIL6-F: GCTACCAAACTGGATATAATCAGGA, mIL6-R: CCAGGTAGCTATGGTACTCCAGAA; COX2-F: CTTCGGGAGCACAACAGAG; COX2-R: GCGGATGCCAGTGATAGAG. Relative gene expression was calculated with the delta delta CT method using a software (CFX Maestro™, BioRad, Hercules, CA, USA). Reactions were run in duplicates. The supernatant was analyzed for IL6 secretion by immunoassay according to the manufacture’s instruction (R&D Systems, Minneapolis, MN).

### 4.5. Endotoxin Detection Using LAL Test

To evaluate the level of endotoxin in clean and uncleaned ADL, a Limulus amebocyte lysate assay (LAL; Life Technologies, Carlsbad, CA, USA) was performed. LAL was added to the different preparations of ADL, following 10 min of reaction, the colorimetric substrate was added. The reaction was stopped using acetic acid. OD was read at 405 nm.

### 4.6. Immunostaining

Translocation of NFκB-p65 from cytoplasm to the nucleus was detected using an immunofluorescent analysis. RAW 264.7 cells were seeded onto glass slides (Merck, Darmstadt, Germany). Following 1 h stimulation of the cells by respective samples, cells were fixed by paraformaldehyde and blocked with PBS containing 1% BSA and 0.3% Triton at room temperature for 1 h. NFκB-p65 primary antibody (Cell Signaling Technology, Denver, MA, USA) was applied overnight at 4 °C. Following removal of the primary antibody, the secondary antibody labeled with Alexa 488 (CS-4412, Cell Signaling Technology) was added for 1 h at room temperature. Images were captured using a fluorescent microscope (Oxion fluorescence, Euromex, Arnheim, The Netherlands).

### 4.7. Statistical Analysis

The experiments were repeated three to six times. Bar in Figure 1 shows the mean and standard deviation of the cumulative data from all experiments. Statistical analysis was based on Mann–Whitney U test and Kruskal–Wallis test without Dunn’s multiple comparisons correction and Friedman test. For the immunoassay, corresponding medians are reported. Analyses were performed using Prism v8 (GraphPad Software, La Jolla, CA, USA). Significance was set at *p* < 0.05.
